# Prevalence of intestinal parasitic infections among highland and lowland dwellers in Gamo area, South Ethiopia

**DOI:** 10.1186/1471-2458-13-151

**Published:** 2013-02-18

**Authors:** Teklu Wegayehu, Tsegaye Tsalla, Belete Seifu, Takele Teklu

**Affiliations:** 1 Department of Biology, Arba Minch University, P. O. Box 21, Arba Minch, Ethiopia; 2 PhD candidate in Tropical and Infectious Diseases, Aklilu Lemma Institute of Pathobiology, Addis Ababa University, Addis Ababa, Ethiopia; 3 Department of Medical Laboratory Science, Arba Minch University, P. O. Box 21, Arba Minch, Ethiopia; 4 School of Biomedical and Laboratory Sciences, University of Gondar, P. O. Box 196, Gondar, Ethiopia

**Keywords:** Control strategies, Intestinal parasites, Parasitism, Protozoan

## Abstract

**Background:**

Epidemiological information on the prevalence of intestinal parasitic infections in different regions is a prerequisite to develop appropriate control strategies. Therefore, this present study was conducted to assess the magnitude and pattern of intestinal parasitism in highland and lowland dwellers in Gamo area, South Ethiopia.

**Methods:**

Community-based cross-sectional study was conducted between September 2010 and July 2011 at Lante, Kolla Shelle, Dorze and Geressie kebeles of Gamo Gofa Zone, South Ethiopia. The study sites and study participants were selected using multistage sampling method. Data were gathered through house-to-house survey. A total of 858 stool specimens were collected and processed using direct wet mount and formol-ether concentration techniques for the presence of parasite.

**Results:**

Out of the total examined subjects, 342(39.9%) were found positive for at least one intestinal parasite. The prevalence of *Entamoeba histolytica/dispar* was the highest 98(11.4%), followed by *Giardia lamblia* 91(10.6%), *Ascaris lumbricoides* 67(7.8%), *Strongyloides stercoralis* 51(5.9%), hookworm 42(4.9%), *Trichuris trichiura* 24(2.8%), *Taenia* species 18(2.1%), *Hymenolepis nana* 7(0.6%) and *Schistosoma mansoni* 1(0.12%). No statistically significant difference was observed in the prevalence of intestinal parasitic infections among lowland (37.9%) and highland dwellers (42.3%) (P = 0.185). The prevalence of intestinal parasitic infection was not significantly different among the study sites but it was relatively higher in Geressie (42.8%) than other kebeles. Sex was not associated with parasitic infections (P = 0.481). No statistically significant difference of infection was observed among the age groups (P = 0.228) but it was higher in reproductive age group.

**Conclusions:**

The high prevalence of intestinal parasitic infections among the lowland and highland dwellers in Gamo area indicated that parasitic infections are important public health problems. Thus, infection control measures and the development of awareness strategies to improve sanitation and health education should be considered.

## Background

Intestinal parasitic infestation represents a large and serious medical and public health problem in developing countries. It is estimated that some 3.5 billion people are affected, and that 450 million are ill as a result of these infections, the majority being children [1]. Apart from causing morbidity and mortality, infection with intestinal parasites has known to cause iron deficiency anemia, growth retardation in children and other physical and mental health problems [2]. Furthermore, chronic intestinal parasitic infections have become the subject of speculation and investigation in relation to the spreading and severity of other infectious diseases of viral origin, tuberculosis and malaria [3-6].

Several factors like climatic conditions, poor sanitation, unsafe drinking water, and lack of toilet facilities are the main contributors to the high prevalence of intestinal parasites in the tropical and sub-tropical countries [7]. In addition, intestinal parasitic agents increase in polluted environments such as refuse heaps, gutters and sewage units in and around human dwelling and living conditions of the people in crowded or unhealthy situations [8]. Hence, a better understanding of the above factors, as well as how social, cultural, behavioral and community awareness affect the epidemiology and control of intestinal parasites may help to design effective control strategies for these diseases [9,10].

Intestinal parasites are widely distributed in Ethiopia largely due to the low level of environmental and personal hygiene, contamination of food and drinking water that results from improper disposal of human excreta [11,12]. In addition, lack of awareness of simple health promotion practices is also a contributing factor [13]. According to the Ethiopian Ministry of Health [14] more than half a million annual visits of the outpatient services of the health institutions are due to intestinal parasitic infections. However, this report may be an underestimate, because most of the health institutions lack appropriate diagnostic methods to detect low levels of parasite burden. In addition, some of the diagnostic methods for specific intestinal parasites, especially for the newly emerging opportunistic intestinal parasites, are not available to peripheral health institutions.

Previous studies gave due attention to the distributions of intestinal parasites in different altitudes, community groups such as school children or other groups confined to camps [15-18]. Hence, the pattern of intestinal parasitism in a community with diverse groups of people as a whole was not illustrated particularly in the study area. The purpose of this study was to assess the magnitude and patterns of intestinal parasitism in highland and lowland dwellers, Gamo area, South Ethiopia.

## Methods

### Study area

A community-based cross-sectional study was conducted between September 2010 and July 2011 in four selected areas of Gamo Gofa Zone administrative sub-division: Lante, Kolla Shelle, Dorze and Geressie kebeles. The zone is located at 505 kms South of Addis Ababa and has a total area of 12, 581.4 square kms. The general elevation of the zone ranges from 600 to 3300 m above sea level. The highland and lowland areas of the zone are characterized by an average annual rain fall of 1166 mm and 900 mm, respectively. The topography of the land is characterized by an undulating feature that makes the existence of different climatic zones in the area possible.

Two of the study ‘kebeles’ (these are the smallest officially acknowledged administrative vicinities in the zone): Dorze and Geressie kebeles are positioned on the highland areas where as Lante and Kolla Shelle kebeles are situated on lowland settings of the zone. They are located a short distance from the zonal administrative center, Arba Minch. Dorze and Lante are located at 30 kms and 22 kms to the north, where as Geressie and Kolla Shelle are located at 56 kms and 28.6 kms to the south of Arba Minch, respectively. In all study kebeles healthcare is provided by health centers and private clinics which are staffed by few health officers, nurses and laboratory technicians. Though it is irregular and non-continuous, the control measures for intestinal parasites include health education, deworming of under five year children and treatment of drinking water.

### Sample size and sampling techniques

The sample size was determined using the single proportion population formula. It was calculated based on a prevalence of 83% [19] with a margin of error of 0.05 and a confidence level of 95%. The design effect was calculated by taking the intraclass correlation for the statistic (i.e.1%) in parasitic infection in highland and lowland areas of Ethiopia. A design effect of 4 was used to allow for multistage sampling. The calculated study sample size was 867. The study sites were divided into two regions based on altitude and from each altitude two kebeles were randomly selected. The households were selected using systematic sampling method and the study individuals were chosen using a simple random sampling method. The calculated sample size was divided to each kebele based on population size. As a result, 464 (192 from Lante and 272 from Kolla Shelle) samples were obtained from lowland and 403 (124 from Dorze and 279 from Geressie) samples were obtained from highland settings.

### Stool collection and processing

About 2 g of fresh fecal samples were collected from each consenting study subject and placed in separate labeled clean plastic stool containers. At the time of collection, date of sampling, the name of the participant, age, sex and consistency of the stool (formed, soft, semi-soft and watery) were recorded for each subject on a recording format. A portion of stool was examined at field by direct wet mount with saline (0.85% sodium chloride solution) to observe motile intestinal parasites and trophozoites under light microscope at 100× and 400× magnifications. The remaining part was preserved with 10% formalin in the ratio of 1 g of stool to 3 ml of formalin for later examination at Arba Minch University. Lugol’s iodine staining technique was also done to observe cysts of the intestinal protozoan parasites. A portion of preserved stool sample was processed by formol-ether concentration method as described by Ritchie [20], with some modification. In brief the stool sample was sieved with cotton gauze and transferred to 15 ml centrifuge tube. Then 8 ml of 10% formalin and 3 ml of diethyl ether was added and centrifuged for 2 min at 2000 rpm. The supernatant was discarded and the residues were transferred to microscopic slides and observed under light microscope at 100× and 400× magnifications for the presence of cysts and ova of the parasites. The presence of parasites was confirmed when observed by any of the methods above.

### Quality control

Before starting the actual work, quality of reagents and instruments were checked by experienced laboratory technologist. The specimens were also checked for serial number, quality and procedures of collection. To eliminate observer bias, each stool sample was examined by two laboratory technicians. The technicians were not informed about the health and other status of the study participants. In cases where the results were discordant, a third senior reader was used. The result of the third expert reader was considered the final result.

### Data analysis

Statistical analysis was performed with SPSS software version 16. Chi-square (*χ*2) was used to verify possible association between infection and exposure to different factors. Probability values were considered to be statistically significant when the calculated P-value was equal to or less than 0.05.

### Ethical clearance

The study was reviewed and approved by ethical committee of Arba Minch University. The ethical considerations were addressed by treating positive individuals using standard drugs under the supervision of a local nurse. The objective of the study was explained to kebele leaders and dwellers; and written consent was sought from parents or guardians of the selected children during stool sample collection.

## Results

A total of 867 study participants were selected for investigation. However, 10(1.2%) were excluded because of inability to provide specimen. For this reason a total of 858 individuals were included in the study (Table 1). Three hundred forty two of the study individuals were found to have single or multiple intestinal parasitic infections, which make the overall prevalence 39.9%. Of the entire positive samples for the parasite, 188 were female participants and 154 were male participants with female to male ratio of 1:0.8 (Table 1). The mean age of the participants was 25 ± 19.

**Table 1:** Frequency distribution of sex, age group and altitude of the study subjects in Gamo area

**Characteristics**	**Frequencies**			***Χ***^**2**^	**P value**
	**Positive (%)**	**Negative**	**Total**		
**Sex**					
Male	154 (38.6)	245	399		
Female	188 (41.0)	271	459	0.49	0.481
**Total**	**342 (39.9)**	**516**	**858**		
**Age group**					
< 4	20 (29.4)	48	68		
5-14	91 (38.6)	145	236		
15-44	179 (42.3)	244	423	4.33	0.228
> 44	52 (39.7)	79	131		
**Total**	**342 (39.9)**	**516**	**858**		
**Altitude**					
Lowland*	174 (37.9)	285	459		
Highland**	169 (42.3)	230	399	1.75	0.185
**Total**	**342 (39.9)**	**516**	**858**		

Key: * Lante and Kolla Shelle.

** Dorze and Gereessie.

**Figure 1:**
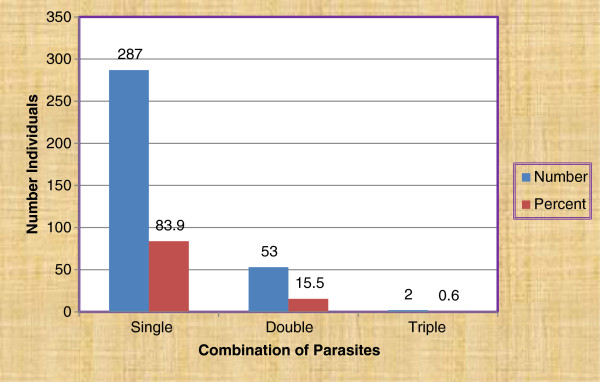
Single and mixed infections among residents of Gamo area, Gamo Gofa Zone, South Ethiopia.

Different types of parasites including protozoans, trematode, cestodes and nematodes were detected from the stool samples of study participants. Prevalence of *Entamoeba histolytica/dispar* (*E. histolytica/dispar*) was the highest 98(11.4%), followed by *Giardia lamblia* (*G. lamblia*) 91(10.6%), *Ascaris lumbricoides* (*A. lumbricoides*) 67(7.8%), *Strongyloides stercoralis* (*S. stercoralis*) 51(5.9%), hookworm 42(4.9%), *Trichuris trichiura* (*T. trichiura*) 24(2.8%), *Taenia* species (*Taenia* spp.) 18(2.1%), *Hymenolepis nana* (*H. nana*) 7(0.6%) and *Schistosoma mansoni* (*S. mansoni*) 1(0.12%), in that order. The majorities of the positive cases were single infections (83.9%) and double infections (15.5%). Of the triple infected persons, one was coinfected with *E. histolytica/dispar, G. lamblia* and *Taenia* spp. and the other with *E. histolytica/dispar, S. stercoralis* and hookworm (Figure 1).

The prevalence of infection with different intestinal helminths and protozoan parasites for lowland (Lante and Kolla Shelle) and highland (Dorze and Gressie) is shown in Table 2. Out of 459 stool samples collected from lowland area, 174(37.9%) were positive for at least one parasite. Similarly, of the 399 stool samples collected from highland area, 169(42.3%) were positive for at least one parasite. No statistically significant difference was observed (P = 0.185) between presence of intestinal parasites and altitude (Tables 1 and 2). However, *G. lamblia* (P < 0.001) and hookworm (P = 0.002) were significantly more prevalent in lowland areas whereas *A. lumbricoides* (P < 0.001) and *T. trichiura* (P < 0.001) were significantly more prevalent in highland areas, but *S. stercoralis* was exclusive to lowland areas (Table 2).

**Table 2:** Prevalence of intestinal parasites among study subjects in lowland and highland dwellers in Gamo area

**Parasites**	**Study sites**						**Total (n = 858) No (%)**	***χ***^**2**^	**P-value**
	**Lowland**			**Highland**					
	**Lante (n = 190)**	**Shelle (n = 269)**	**Total (n = 459)**	**Dorze (n = 123)**	**Geressie (n = 276)**	**Total (n = 399)**			
	**No (%)**	**No (%)**	**No (%)**	**No (%)**	**No (%)**	**No (%)**			
*E. histolytica/dispar*	9 (4.7)	19(7.1)	28 (6.1)	1 (0.8)	69 (25.0)	70 (17.5)	98 (11.4)	27.63	< 0.001*
*G. lamblia*	28 (14.7)	46 (17.1)	74 (16.1)	2 (1.6)	15 (5.4)	17 (4.3)	91 (10.6)	31.67	<0.001*
*A. lumbricoides*	2 (1.1)	1 (0.4)	3 (0.6)	27 (21.9)	37 (13.4)	64 (16.0)	67 (7.8)	70.19	<0.001*
*H. nana*	2 (1.1)	3 (1.1)	5 (1.1)	1 (0.8)	1 (0.4)	2 (0.5)	7 (0.6)	0.91	0.344
Hookworm	14 (7.4)	19 (7.0)	33 (7.2)	1 (0.8)	8 (2.9)	9 (2.2)	42 (4.9)	9.83	0.002*
*S. mansoni*	1 (0.5)	----	1 (0.2)	----	----	-----	1 (0.12)	--	---
*S. stercoralis*	17 (8.9)	34 (12.6)	51 (11.1)	----	----	-----	51 (5.9)	--	---
*Taenia spp*	4 (2.1)	9 (3.3)	13 (2.8)	4 (3.3)	1 (0.4)	5 (1.2)	18 (2.1)	2.59	0.107
*T. trichiura*	----	1 (0.4)	1 (0.2)	21 (17.0)	2 (0.7)	23 (5.8)	24 (2.8)	24.21	<0.001*

Key: *χ*^2^ and P-value values represent lowland and highland comparison.

* Represent statistically significant difference (P < 0.05).

As observed in Table 2, of 190 and 269 stool samples collected from Lante and Kolla Shelle, 66(34.7%) and 108(40.1%) were found positive for at least one parasite, respectively. Similarly, out of 123 and 276 fecal samples collected from Dorze and Geressie sites, 50(40.7%) and 118(42.8%) were infected with one or more intestinal parasites. The overall difference in parasite prevalence was no statistically significant among the study sites, (P = 0.38). However, *E. histolytica/dispar* prevalence was significantly higher in Geressie (P < 0.001) than other study areas. *A. lumbricoides* and *T. trichiura* were significantly higher in Dorze (P < 0.005) and *S. mansoni was* detected in Lante alone*.*

The distribution of infection among female and male participants is shown in Table 3. Female participants showed the highest infection rate (41.0%), followed closely by male participants (38.6%) (Table 1). The calculated P-value (0.48) indicates that the difference in the prevalence of intestinal parasites between female and male participants was statistically not significant. However, *A. lumbricoides* infection was significantly higher (P = 0.004) among female participants than male participants (Table 3).

**Table 3:** Sex related prevalence of intestinal parasites among study subjects in Gamo area

**Parasites detected**	**Sex**		***χ***^**2**^	**P-value**
	**Male (n = 399)**	**Female (n = 459)**		
	**No (%)**	**No (%)**		
*E. histolytica/dispar*	37 (9.3)	61 (13.3)	3.40	0.065
*G. lamblia*	43 (10.8)	48 (10.5)	0.02	0.880
*A. lumbricoides*	20 (5.0)	47 (10.2)	8.10	0.004^*^
*H. nana*	4 (1.0)	3 (0.7)	0.32	0.571
Hookworm	25 (6.3)	17 (3.7)	3.01	0.083
*S. mansoni*	1 (0.3)	-----	--	---
*S. stercoralis*	28 (7.0)	23 (5.0)	1.54	0.215
*Taenia spp*	8 (2.0)	10 (2.2)	3.62	0.057
*T. trichiura*	10 (2.5)	14 (3.1)	0.23	0.630

Key: * Represent statistically significant difference (P < 0.05).

To see variations in age group, the study population was divided into 4 age groups: birth to 4 years, 5 to 14 years, 15–44 years, and over 44 years. The distribution of infection among study subjects in the different age groups is shown in Table 4. The overall infection rate was highest among the 15–44 years age group (42.3%) followed by above 44 years age group (39.7%). Only 29.4% of children from birth to 4 years age group were infected. The difference in the prevalence of intestinal parasites was insignificant (P = 0.228) among the age groups (Table 1). Each parasite was also not associated with the age of the participant (P > 0.005) except *T. trichiura* which shown significantly high (P = 0.007) prevalence among the age group of 5–14 years (Table 4).

**Table 4:** Age related prevalence of intestinal parasites among study subjects in Gamo area

**Parasites detected**	**Age Group**				***χ***^**2**^	**P-value**
	**< 4 (n = 68)**	**5-14 (n = 236)**	**15-44 (n = 423)**	**> 44 (n = 131)**		
	**No (%)**	**No (%)**	**No (%)**	**No (%)**		
*E. histolytica/dispar*	9 (13.2)	21 (8.9)	54 (12.8)	54 (12.8)	2.56	0.462
*G. lamblia*	10 (14.7)	22 (9.3)	22 (9.3)	15 (11.5)	1.73	0.630
*A. lumbricoides*	2 (2.9)	22 (9.3)	29 (6.9)	14 (10.7)	5.03	0.170
*H. nana*	----	4 (1.7)	3 (0.7)	----		----
Hookworm	-----	10 (4.2)	24 (5.7)	8 (6.1)	4.68	0.197
*S. mansoni*	----	----	1 (0.2)	------		-----
*S. stercoralis*	1 (1.5)	13 (5.5)	26 (6.1)	11 (8.4)	3.96	0.266
*Taenia spp*	-----	-----	16 (3.9)	2 (1.5)		----
*T. trichiura*	1 (1.5)	14 (5.9)	6 (1.4)	3 (2.3)	12.05	0.007*

Key: < 4 years = Preschool children, 5–14 years = School children, 15–44 years = Reproductive age, > 44 years = elder.

* Represent statistically significant difference (P < 0.05).

## Discussion

Primary objectives of epidemiological studies on the prevalence of infection of intestinal parasites in different regions/localities are to identify high-risk communities and formulate appropriate intervention. In line with this view, the present study attempted to assess the prevalence of different intestinal parasitic infections in highland and lowland dwellers in Gamo area, Gamo Gofa Zone and then recommend appropriate intervention.

The results of the study showed the occurrence of several intestinal parasites of public health importance among inhabitants in four kebeles found in Gamo area of Gamo Gofa Zone, South Nations. The overall prevalence of 39.9% with one or more intestinal parasites found in this study was much lower than what was reported (82.8%) from residents of four villages in southwestern Ethiopia by Yeneneh [21]; (83.8%) from school-children around Lake Langano by Legesse and Erko [22] and from that of Mengistu and collegues [19] (83%) from urban dwellers in southwest Ethiopia. However, the prevalence in our study was slightly higher compared to other community-based studies conducted in Saudi Arabia by Al-Shammari *et al.*[23] showing an overall prevalence of 32.2%. The possible explanations for the discrepancy between the present and previous study finding might be the result of variation in sampling techniques used, the difference in the quality of drinking water source, and variation in the environmental condition of the different study localities.

The prevalence of *E. histolytica/dispar* and *G. lamblia* infection in this study was 11.4% and 10.6%, respectively. These are within the range of the nation-wide prevalence rate for amoebiasis and giardiasis [24]. However, the present finding was relatively higher than that reported from urban dwellers in southwest Ethiopia, where Mengistu *et al.*[19] recorded 3.1% and 3.6% for amoebiasis and giardiasis, respectively [19]. The prevalence of giardiasis was also higher than that of Birrie and Erko report of 3.1% among non-school children [25]. *Giardia* cysts have been isolated from water supplies in different parts of the world [26,27]. Epidemic giardiasis may be related to drinking water [28]. The present study was also conducted in a rural area that may share the mentioned risk factors.

The level of ascariasis observed in this study (7.8%) of fecal samples was far lower than that reported from urban dwellers in Jimma, where Mengistu *et al.*[19] recorded 41.0% prevalence. The prevalence of hookworm infection was 42(4.9%). The rate is lower than the previous community based study in Jimma, where Mengistu and colleagues recorded 17.5% prevalence [19]. However, the present findings on prevalence of *S. stercoralis*, *H. nana*, and *Taenia* spp. was not much different from the findings of previous studies reported by Woldemichael *et al.,* McConnel and Armstrong [18,24].

Although significant difference was not observed, the prevalence of intestinal parasites infections was slightly lower among lowland dwellers (37.9%) than highland dwellers (42.3%). The existence of relatively low prevalence of human intestinal parasites in lowlands is due to low prevalence of *A. lumbricoides and T. trichiura*. A nation-wide study conducted on ascariasis in Ethiopia has indicated a low prevalence of ascariasis in the low and dry areas of the country [29]. In agreement to this report, the present study showed relatively low prevalence of ascariasis (0.7%) and trichuriasis (0.2%) among lowland dwellers. This might be because of proper toilet facilities, apposite hand washing habit and better awareness about health in lowland dwellers than highland dwellers in our study area.

Though the reason for high prevalence of *G. lamblia* in lowland area is unclear, the high prevalence of hookworm infection might be contributed to low shoe wearing habit during irrigation. According to the geo climatic type of area, significant highest prevalence of *S. stercoralis* infection was found in lowland. The explanation of this is that prevalence of *S. stercoralis* infection is parallel with hookworm infection [30]. This may lead us to a conclusion that geo climatic factors, such as soil textures, farming ecosystem, temperature, humidity and rainfall essentially influence the infection of *S. stercoralis* as the lowlands are located near Lake Abaya.

From the four study sites, *S. mansoni* was reported only from Lante kebele. However, the prevalence of *S. mansoni* (0.12) was much lower as compared to 14.8% reported by Mengistu *et al.*[19]. Although this study did not evaluate the migratory history of the study participants, the existence of such low level infection rate may imply the relocation of infected individual from endemic foci. Nevertheless, the presence of schistosome-infected dweller in Lante kebele represents a risk for the introduction of a new transmission focus where the snail hosts might be available. Hence, extensive study on the transmission foci in the area is recommended to take timely intervention.

Variations that might have occurred due to gender and age group differences were also examined in this current study. Variation of results due to gender differences was not observed in the study. The result was similar to a study conducted in the central part of Turkey [31]. But *A. lumbricoides* infection was significantly higher among female participants than male participants in this study. Though significant difference among age groups was not observed in the current study, the prevalence of infection was higher in reproductive age group. This might be due to the fact that this is the age group of working people who are engaged in agriculture and most likely exposed to infection through contaminated soil, water and food. The rate of infection by *Taenia* spp. was also higher in 15–44 and > 44 age groups. This could be due to the fact that as the child grows older the habit of feeding beef increases.

Multiple infections occurred in 55 individuals making 6.4% of the total examined subjects and 16.1% of those who had intestinal parasites. The level of double infections with intestinal parasites determined in the present study (6.2%) was much lower than what was reported from southwest Ethiopia portraying a double infection of 35.8% among urban communities [19]. The possible difference in the socio-demographic condition of the study population and the environmental condition might explain the observed difference in double infection in the two study localities.

This study did not assess opportunistic intestinal parasitic infections due to lack of laboratory facilities in our department. Although important risk factors such as age, sex and altitude were considered, some risk factors were not evaluated in the current study. Apart from these limitations our study has the following strengths. It is the first of its kind in the area i.e. pattern of intestinal parasitism was not studied earlier than this current study in the study area. Moreover, all the participants from each kebele were sampled at one specific time in the sampling period to avoid seasonal biases. In addition, the sample was collected from the entire population of highland and lowland dwellers by giving equal probability for each individual in selected kebeles which reflects the real prevalence of intestinal parasites in Gamo area. Furthermore, standard laboratory techniques were used; all laboratory tasks followed standard procedures; and quality control mechanisms were employed at each step of the study.

## Conclusion

The high prevalence of intestinal parasitic infections among the lowland and highland dwellers in Gamo area indicated that parasitic infections are considerable public health problems. The present study has also revealed that *E. histolytica/dispar* and *Giardia* as common protozoan while *A. lumberciod*, hookworm and *T. trichiura* as common helminths that cause parasitic infection with varying magnitude in the study area. Enhancing socioeconomic status, improving sanitation facilities, instilling health education and promoting ways of keeping personal hygiene can be good strategies to control these infections in the area.

## Competing interests

The authors declare that they have no competing interests.

## Authors’ contributions

TW Conception of the research idea, designing, collection of data, data analysis, interpretation, and manuscript drafting. TT^1^ Designing, collection of data and manuscript reviewing. BS Collection of data and manuscript reviewing. TT Data analysis, interpretation and manuscript drafting. All authors read and approved the final manuscript.

## Pre-publication history

The pre-publication history for this paper can be accessed here:

http://www.biomedcentral.com/1471-2458/13/151/prepub
